# Association of Health Care Use and Economic Outcomes After Injury in Cameroon

**DOI:** 10.1001/jamanetworkopen.2020.5171

**Published:** 2020-05-19

**Authors:** S. Ariane Christie, Drusia Dickson, Susana N. Mbeboh, Frida N. Embolo, William Chendjou, Emerson Wepngong, Ahmed N. Fonje, Eunice Oben, Kareen Azemfac, Alain Chichom Mefire, Theophile Nana, M. Agbor Mbianyor, Patrick Stern, Rochelle Dicker, Catherine Juillard

**Affiliations:** 1 Department of Surgery, University of California, San Francisco; 2 Faculty of Health Sciences, University of Buea, Buea, Cameroon; 3 Department of Surgery, Limbe Regional Hospital, Limbe, Cameroon; 4 Department of Surgery, UCLA (University of California, Los Angeles)

## Abstract

**Importance:**

Despite the highest injury rates worldwide, formal medical care is not often sought after injuries in Sub-Saharan Africa. Unaffordable costs associated with trauma care might inhibit injured patients from seeking care.

**Objectives:**

To (1) determine the injury epidemiology in Cameroon using population-representative data, (2) identify the barriers to use of formal health care after injury, and (3) determine the association between use of care and economic outcomes after injury.

**Design, Setting, and Participants:**

This mixed-methods, cross-sectional study included a population-representative, community-based survey and nested qualitative semistructured interviews in the urban-rural Southwest Region of Cameroon. Three-stage cluster sampling was used to select target households. Data were collected from January 3 to March 14, 2017, and analyzed from March 3, 2017, to March 3, 2019.

**Exposures:**

Injuries occurring in the preceding 12 months.

**Main Outcomes and Measures:**

Postinjury use of health care services, disability, and economic outcomes. All survey data were adjusted for cluster sampling.

**Results:**

Of 1551 total households approached, 1287 (83.0%) were surveyed for a total sample size of 8065 participants. The 8065 individuals surveyed included 4181 women (52.0%), with a mean age of 23.9 (standard error [SE], 0.2) years. A total of 503 injuries were identified among 471 unique participants, including 494 nonfatal injuries. Among these, 165 (34.6%) did not seek formal medical services. Disability occurred after 345 injuries (68.6%) and resulted in 11 941 lost days of work in the sample. Family economic hardship after injury was substantially increased among the injured cohort who used formal medical care. Injuries brought to formal medical care, compared with those that were note, incurred higher mean treatment costs ($101.08 [SE, $236.23] vs $12.13 [SE, $36.78]; *P* < .001), resulted in higher rates of lost employment (19.9% [SE, 3.6%] vs 5.6% [SE, 1.6%]; *P* = .004), and more frequently led affected families to use economic coping strategies, such as borrowing money (26.2% [SE, 2.7%] vs 7.1% [SE, 1.2%]; *P* < .001). After adjusting for age and severity, use of formal medical care in Cameroon was independently associated with severe economic hardship after injury, defined as a new inability to afford food or rent (adjusted odds ratio, 1.67; 95% CI, 1.05-2.65).

**Conclusions and Relevance:**

In this study, injury in Southwestern Cameroon was associated with significant disability and lost productivity. Formal medical treatment of injury was associated with significant financial consequences for households of injured patients. Primary prevention of road traffic injuries and financial restructuring of emergency care could improve trauma care access in Cameroon and reduce the societal effects of injury.

## Key Points

**Question:**

What is the association between use of medical care and financial outcomes after injury in a central sub-Saharan African country?

**Findings:**

In this population-representative, cross-sectional, mixed-methods study of 8065 Cameroonians, cost was perceived as a major barrier to seeking formal medical care after injury. Family economic hardship was substantially increased among the injured cohort who used formal medical care.

**Meaning:**

The prohibitive expense associated with formal medical care in Cameroon was associated with care rationing and financial instability after injury; cost restructuring of injury care may be necessary to increase formal use of health care and improve injury outcomes.

## Teaser

This cross-sectional survey and qualitative study assesses injury epidemiology in Cameroon using population-representative data, identifies the barriers to use of formal health care, and determines the association between use of care and economic outcomes among participants in a community-based survey.

## Introduction

An estimated 77% of preventable deaths associated with inadequate surgical care are attributable to injuries, and 90% of injury-related deaths worldwide occur in low- and middle-income countries (LMICs).^1,2^ In many of these countries, use of formal medical services after injury can require potentially crippling out-of-pocket expenditures. The long-term economic implications of injury in LMICs, however, remain largely unknown.^2,3^ Efforts to expand access to trauma care have been limited by the lack of injury epidemiology data and a poor understanding of barriers to use of care.

Historically, most data on injury in LMICs were extracted from retrospective hospital and police records, which are subject to selection bias and underestimate the burden of injury. Trauma registries are essential to injury surveillance but fail to capture data on persons who do not use formal medical care, likely excluding the most vulnerable populations. A pilot trauma registry in Cameroon^4^ found that persons from the lowest socioeconomic strata were underrepresented in hospital trauma cohorts despite increased injury severity. As in many sub-Saharan African nations, injury treatment in Cameroon is based on fee for service, raising concerns that cost considerations constitute major barriers to injury care.

We hypothesized that injury in Cameroon results in catastrophic economic consequences for a significant proportion of the population and that these consequences are worse for those who seek formal medical care. This study aimed to (1) estimate the epidemiological burden associated with injury (incidence and disability), (2) identify barriers and facilitators for the use of formal care after injury, and (3) identify factors associated with economic hardship after injury.

## Methods

### Study Design, Survey Setting, and Sampling Framework

We performed a cross-sectional, mixed-methods study consisting of a community-based survey on injury and a nested qualitative study on care-seeking behavior after injury in the Southwest Region of Cameroon. The region has a mixed urban-rural distribution with a combined population of 1 575 224.^5^ A 3-stage clustered sampling framework was used to select 32 distinct sampling areas. The first stage consisted of probability-proportionate-to-size sampling that was used to select 9 health districts. The second stage included 4 health areas per district. Finally, in the third stage, a random starting point within each health area was selected using geolocation data to identify a starting household. Contiguous households were approached from that point until the target sample at each site was reached (n = 200). Two Southwestern districts, Akwaya and Bakassi, were excluded from the sampling framework owing to travel barriers in these regions. Ethical approval was obtained from the institutional review board of the University of California, San Francisco, and University of Douala, Douala, Cameroon, and all participants provided oral informed consent. Reporting guidelines from the American Association for Public Opinion Research (AAPOR) were followed to determine eligibility and calculate survey outcome rates for in-person household surveys.^6^

Sample size targets were estimated using the limited existing data from prior community-based surveys in Sub-Saharan Africa that estimate injury incidence from 32.0 (95% CI, 29.9-35.7) to 100.0 (95% CI, 91.4-106.9) per 1000 person-years.^7,8^ Therefore, a minimum sample size of 4680 was calculated to provide 78% power to detect an estimated 6% yearly incidence of injury with a precision of plus or minus 5% and a 95% CI, adjusted for 20% estimated nonresponse and a design effect of 2 for multicluster sampling.^9^ Because our broader community-based survey included multiple subanalyses of rare events, the sample size was deliberately exceeded during collection by 50% at each site to maintain a representative sample.

### Household Surveys

Data were collected during an 8-week period from January 3 to March 3, 2017. To minimize reporting bias, Cameroonian nationals (S.N.M., F.N.E., W.C., E.W., A.N.F., E.O., K.A., and M.A.M.) were selected as research assistants and trained in surveying techniques and qualitative interviewing. Targeted households were asked to identify a family representative (aged ≥18 years), and consent was obtained using a standard oral consent script.

After consent was obtained, research assistants (S.N.M., F.N.E., W.C., E.W., A.N.F., E.O., K.A., and M.A.M.) verbally administered a pilot-tested survey addressing all members of the household. Information collected included demographic data on each individual, household economic indicators,^10^ and household perceptions of access to and quality of medical care. Family representatives were asked to identify members of the household, living or deceased, who sustained an injury within the past 12 months.^11^ *Injury* was defined as any sudden bodily insult directly resulting in death or loss of routine activity by any family member for at least 1 day or that required medical care, regardless of whether such care was obtained.^12^ For each injury identified, additional information on injury characteristics, care-seeking behavior, injury treatment, disability, and economic effects was requested (eAppendix in the Supplement). Severity indicators were included in the survey as a surrogate method of identifying severe injuries, including cessation of breathing, disorientation or amnesia to the event, or loss of consciousness.

### Qualitative Interviews

A standard, semistructured interview template based on the access-to-care framework developed by Peters et al^13^ was designed to elicit participant-driven discussion regarding perceptions of formal and informal medical care services and to explore determinants of the care decision-making process after injury. Across sampling sites, interview participants were selected for maximal difference with regard to urban-rural community, age, injury characteristics, and care-seeking choices. Interviews were performed in the participant’s preferred language and recorded for cross-validation. Interviews were conducted until thematic saturation was noted during coding. All interviews ultimately conducted were coded and included in the analysis.

### Data Management and Analysis

Data were analyzed from March 3, 2017, to March 3, 2019. Paper survey forms were securely stored, and the data were manually entered into an encrypted REDCap database.^14^ All statistical analyses were performed in STATA, version 14 (StataCorp LLC)^15^ and adjusted for clustered sampling methods using the *svy* command. Descriptive analyses of care-seeking cohorts included proportions, means, and standard errors (SEs) for normally distributed variables and medians and interquartile ranges (IQRs) for nonparametric variables. We used χ^2^, adjusted Wald, or Kruskal-Wallis tests as appropriate. Multivariate logistic regression was used to identify factors associated with care-seeking decisions and economic outcomes using a stepwise regression process. For all analyses, a double-sided α level to determine statistical significance was set at .05 and used to calculate *P* values.

Qualitative interviews were transcribed by Cameroonian nationals (E.O., F.N.E., E.W., W.C., and A.N.F.) and analyzed by 2 coders (S.A.C., D.D.) for major themes using deductive and inductive elements. Emerging themes were identified and developed with ongoing reexamination of the data by both coders with Cameroonian interviewer adjudication. Thematic discrepancies^16^ were analyzed with local collaborators to establish clear definitions for all coding elements, and main themes regarding the care decision-making process were summarized. All qualitative analysis of interview transcripts was performed using ATLAS.ti, version 5 (ATLAS.ti Scientific Software Development GmbH).^17^

## Results

### Injury Epidemiology

The study population had a mean (SE) age of 23.9 (0.2) years and included 4181 (52.0%) women and 3865 (48.0%) men. Of 1551 total households approached, 1287 (83.0%) were surveyed for a total sample size of 8065 participants. Data were collected in 15 rural and 18 urban sampling areas throughout the Southwest Region of Cameroon. Demographic indicators were similar to regional data from the 2011 Demographic and Health Survey, although our survey sample reported increased access to cellular telephones (eTable 1 in the Supplement).

Of 8065 participants sampled, 471 (5.8%) sustained 1 or more injury in the past year. Among these individuals, 32 had more than 1 injury in the past year, yielding 503 separate injury events for a total injury incidence of 63 (95% CI, 57-69) injuries per 1000 person-years. Nine fatal injuries were reported for a yearly injury mortality of 113 (95% CI, 65-197) per 100 000 population.

Most injuries occurred in men (309 of 501 [61.7%]) and young persons (mean age, 29.0 [range, 0-95] years). Road traffic injury (RTI) (133 of 484 injuries for which data on mechanism were provided [27.5%]) and falls (120 of 484 [24.7%]) constituted the most common injury mechanisms in all age groups. Seventy-six of 503 injuries in the trauma cohort (15.1%) had 1 or more severity indicators on the day of injury.

### Barriers and Facilitators of Formal Care Utilization

**Figure 1.**
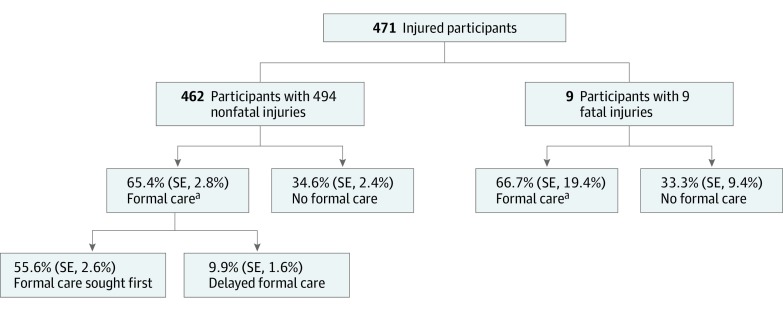
Use of Formal Medical Care After Injury in the Southwest Region of Cameroon Percentages are adjusted for the clustered sampling method. SE indicates standard error. ^a^*P* = .86 for the proportion of formal care seeking among participants with fatal vs nonfatal injuries.

More than one-third of 480 persons with nonfatal injuries who provided information about care-seeking practices (165 [34.6%]) were never treated at formal medical care services, and a further 47 (9.9%) only sought formal medical care after alternative treatment had been attempted (Figure 1). Including 9 fatal injuries, for all of which care-seeking information was available, 44.4% of injured participants did not seek formal care (168 [34.4%]) or sought it only after first seeking alternative care (49 [10.0%]). Home treatments (140 [58.1%]) or consultation with traditional healers (60 [24.9%]) were the most common of the 241 responses on alternative care. Compared with those who sought formal care first, those who sought delayed care and no care were younger (median age, 23 [IQR, 13-39] vs 28 [IQR, 19-40] years) and made less money from their primary job or activity (median weekly wage, 0 [IQR, 0-$17.18] vs $2.97 [IQR, 0-$24.05]) (Table 1). Among the group of 76 participants who reported the presence of 1 or more severity indicators, 18 (23.7%) did not go on to use formal care after first injury.

**Table 1.** Participant and Household Demographics of Participants Who Sought Formal Care vs Those Who Delayed or Did Not Seek Formal Care

Characteristic	Care group^a^		*P* value^b^
	Formal care first (n = 272)	Delayed or no formal care (n = 217)	
Age, median (IQR), y	28 (19-40)	23 (13-39)	.002
Male, % (SE)	61.8 (4.8)	52.0 (5.0)	.14
School completed, median (IQR), y	8 (7-12)	8 (5-13)	.41
Weekly wage, median (IQR), US $^c^	2.97 (0-17.18)	0 (0-17.18)	.04
Urban household, % (SE)	52.5 (14.5)	57.9 (14.6)	.25
Wood cooking fuel used, % (SE)	93.0 (2.4)	91.2 (3.1)	.47
Family owns a cell phone, % (SE)	95.1 (1.7)	95.4 (1.9)	.87
Owns agricultural land, % (SE)	68.2 (5.9)	67.9 (7.0)	.96
Transport time to care, mean (SD), min	21.6 (4.5)	22.8 (6.8)	.71

Abbreviations: IQR, interquartile range; SE, standard error.

^a^ Percentages reported are population estimates that have been adjusted for the clustered sampling method. The SEs present the level of variance.

^b^ Calculated using the χ^2^, adjusted Wald, or Kruskal-Wallis test as appropriate to compare categorical and continuous variables.

^c^ Analysis included individuals who do not earn wages, such as children.

Multivariate logistic regression adjusted for participant age and injury severity identified sustaining injury to the head and/or neck (adjusted odds ratio [AOR], 2.41; 95% CI, 1.33-4.39) or the torso (AOR, 4.61; 95% CI, 1.53-13.95), RTI mechanism (AOR, 2.17; 95% CI, 1.24-3.78), and presence of a nonrelated bystander as a first responder (AOR, 2.65; 95% CI, 1.25-5.60) to be associated with use of formal health care services. Conversely, absence of a first responder (AOR, 1.92; 95% CI, 1.18-3.13) and sustaining a non-RTI blunt trauma mechanism (AOR, 2.42; 95% CI, 1.33-4.41) were associated with delayed or no presentation to formal care (eTable 2 in the Supplement).

Injured participants who delayed use or did not use formal medical care services most often attributed their decision to a belief that their injury was not serious (94 of 216 respondents [43.5%]) or personal preference (69 of 216 [31.9%]), with 25 (11.6%) citing cost as the primary deterrent. Decision-making and care-seeking choices after injury were further explored in 34 semistructured interviews with 39 participants (eTable 3 in the Supplement). Qualitative interviews revealed 4 principal themes regarding the decision-making process after injury: prohibitive cost and cost structure; severity assessment; source of health care beliefs; and acceptance of using multiple sources of care (Table 2).

**Table 2.** Qualitative Summary of Barriers and Facilitators of Use of Formal Care After Injury

Theme	Description	Example
Cost structure as a barrier	Formal clinicians require cash payment in full before treatment is rendered, whereas traditional healers typically allow installation payment and payment in goods.	“When you go to a hospital, they ask you to pay an advance before they start treating you. But with the herbalist…he starts your treatment until a certain duration…But in the hospital, if you don’t have that money, they don’t help.”
		“The nurse is insisting that they will not be attended to if they don’t pay their deposit for bed fee first. This can cause a patient to die easily since they have not been administered any treatment.”
Severity assessment as a barrier or facilitator	Care decisions are heavily influenced by responder evaluations of severity.	“It depends the gravity of the injury. Because let’s say if the, if the oil…instead of falling on his leg, it fell on the whole of his body, we would have taken him to the hospital immediately. Immediately!”
		“You cannot stitch a wound in the house, or a traditional doctor cannot stitch a wound, yes. Or things like, you fell and broke your leg. That is, you feel like the bones are scattered inside. You will just rush to the hospital.”
Source of health care belief as a facilitator	Public health campaigns and advice were cited as influencing the decision-making process. Respondents focused more on advice content rather than expertise of the advice provider.	“And education, [the hospital] gives you education. They educate us, they tell us, ‘this one [illness] isn’t fine’…they take their time to tell us how diseases spread so that we can control them. They help us.”
		“Medical personnel train us. They tell us, ‘If there is an accident and you can’t get a motortaxi quickly, this is what you should give them before you take the person to the hospital.… [Give them] pepper before you take them to the hospital’.… They are trained for both [traditional and formal medicine]. I heard him say he knows both. These Ghanaians, they know plenty about medical herbs.”
Use of multiple forms of care as a barrier or facilitator	Paradigms of care include an acceptance of multiple (sequential or concurrent) use of formal and informal care. Formal care will frequently be used for diagnosis, and then care will be transitioned to traditional medicine for treatment.	“Both [treatments] are fine.… Yes. If there is time for the hospital, we will try the hospital, if it works for you, you are lucky. If there is time for the hospital and it doesn’t work for you, go for the native medicine. If it isn’t the right case for the native doctor, he won’t try, you’ll go to the hospital. Yeah, you jambox [ie, meld or mesh] the two side. Yes.”
		“Well, I just take it maximally, for the hospital is the hospital and…the man in the hospital is doctor, this is to say, is a specialist. And native doctor too is a specialist of different domains.… Yeah, because the doctor can use small machines on you, but with the herbalist, he has his own way of doing his own thing, his own uh, treatment.”

Formal medical services were described in 32 of 34 interviews as prohibitively expensive or openly extortive, whereas nonformal treatments were generally viewed as more economical. In contrast to traditional healers who accept trade or incremental or delayed payments, hospitals and medical centers were described as requiring full payment in advance of consultation. This emphasis on prepayment often degraded community perceptions of the formal medical system as a therapeutic environment. Decisions about care were often determined by participant or responder assessment of severity. Injury assessments were multifactorial and included assumptions about the relative risks of certain injury types or mechanisms. Qualitative respondents cited health care education as an important source of knowledge about injury and a potential benefit of contact with formal care. Respondents did not view use of formal medical care services as a binary alternative. Rather, formal and informal medical care services were most commonly explained as complimentary services to be used in tandem to obtain the best care.

### Disability and Economic Consequences of Injury

Of 503 injury events, 345 (68.6%) resulted in activity-limiting disability as a result of injury (eFigure in the Supplement). Specifically, 160 of 503 injuries (31.8%) resulted in at least 1 severe disability, 130 (25.8%) resulted in moderate disability, and 53 (10.5%) resulted in minor disability only. Data on activity of daily living were obtained for 481 of 503 total injuries. One hundred seventy-eight injuries (37.2%) resulted in the participant being unable to independently perform activities of daily living. In addition, 169 injured participants (35.8%) required another household member to shift their own activities to care for the injured individual. At the time of the study, 114 of 471 injured participants (24.2%) overall remained significantly disabled. Disability after injury led to 11 941 days in the study period during which injured participants or family caretakers were unable to perform their primary jobs or activities owing to the injury (1493 economic disability days per 1000 person-years). Road traffic injuries and falls were the most common mechanisms resulting in lost activity (3834 [32.9%] and 11 659 [29.9%] of all disability days, respectively).

**Figure 2.**
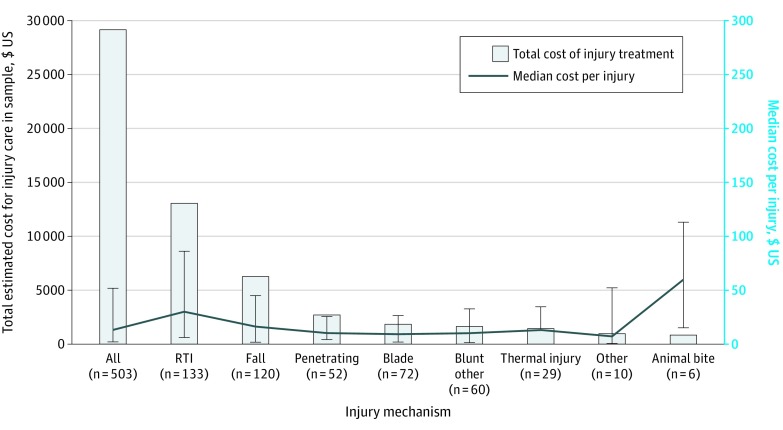
Cost of Injury Treatment in the Southwest Region of Cameroon Includes 471 participants with 503 injuries. Error bars indicate 95% CIs. RTI indicates road traffic injury.

Injury treatment incurred a cost of US $29 145 among study participants, with a median treatment cost of $12.93 per injury (IQR, $1.76- $51.72). Treatment expenditure and cost per injury varied by injury mechanism (Figure 2). Of 484 injuries with both treatment cost and mechanism available, RTI mechanism was responsible for $13 074, or 45.5%, of $28 743 spent on treatment of injury in Cameroon. Treatment costs constituted a mean of 16% (SE, 71%) of injured participants’ annual salaries. Older participants and those from rural areas spent proportionally more of their annual income on treatment than younger and urban cohorts (77.0% of annual salary for those aged ≥60 years [*P* = .01] and 20.1% of annual salary for rural participants [*P* = .03]).

**Figure 3.**
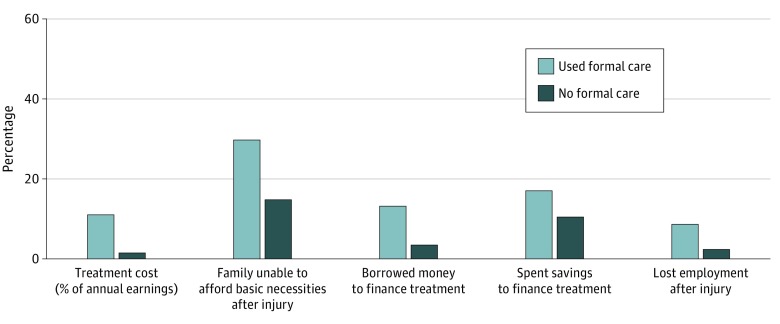
Economic Consequences of Injury by Use of Formal Medical Care in Cameroon Includes 471 participants with 503 injuries. The graph displays reports of economic consequences for the proportion of participants who used vs did not use formal care. *P* < .01 for all comparisons.

Nearly half of all injuries (244 [48.5%]) resulted in the affected household having to spend savings, borrow money, or sell assets to pay for treatment. Data on education and job loss were reported for 490 of 503 injuries. At the time of the survey, 64 participants (13.1%) completely stopped educational activities or lost employment as a direct result of the injury. Data on financial hardship were reported for 475 of 503 injuries. More than one-third of these affected households (163 [34.3%]) reported that after the injury, they experienced severe financial hardship, defined as new inability to afford necessities, including food or housing. For all of these economic indicators, economic disability was markedly worse among the group that used formal care services (Figure 3). Injuries brought to formal medical care incurred higher mean treatment costs ($101.08 [SE, $236.23] vs $12.13 [SE, $36.78]; *P* < .001), resulted in higher rates of lost employment (19.9% [SE, 3.6%] vs 5.6% [SE, 1.6%]; *P* = .004), and more frequently led affected families to use economic coping strategies such as borrowing money (26.2% [SE, 2.7%] vs 7.1% [SE, 1.2%]; *P* < .001). Multivariate logistic regression adjusted for mechanism of injury and disability found the use of formal care to be independently associated with severe financial hardship after injury (AOR, 1.67; 95% CI, 1.05-2.65). Additional independent factors associated with severe financial hardship included disability days (AOR, 1.02; 95% CI, 1.01-1.02), older age (AOR, 1.35; 95% CI, 1.04-1.76), RTI mechanism (AOR, 2.13; 95% CI, 1.29-3.53), rural household (AOR, 2.15; 95% CI, 1.35-3.42), and higher treatment cost (AOR, 1.13; 95% CI, 1.05-1.21) (eTable 2 in the Supplement).

## Discussion

Injury-related mortality in Southwest Cameroon is estimated to be 113 per 100 000 person-years, occurring at double the rate of injury-related death in the United States and exceeding estimated Cameroonian death rates for malaria (45 per 100 000 person-years) and tuberculosis (25 per 100 000 person years) combined.^18,19^ Approximately 44% of injured participants in our study either did not seek formal medical care or sought it only after alternative treatment had been attempted. Both survey and interview data suggested that use of medical care hinges on perceptions of the balance between the economic burden of formal medical treatment and injury severity.

Factors associated with use of health care services were largely related to payment feasibility or responsibility or to severity assessment. Groups less able to finance the high cost of formal medical care had higher rates of delayed care or no care. Similarly, the presence of scene responders without a personal financial stake in the treatment decision was associated with early seeking of formal care. Factors thematically associated with severity, such as RTI, head and/or neck injury, or torso injury were identified as independently associated with use of formal care. However, injured persons or bystanders may fail to recognize severe injuries without external signs of trauma, which may lead to missed severe injury, disability, and death. Ultimately, 23.7% reporting severity indicators failed to seek formal care services, suggesting that community assessment of injury severity likely results in undertriage of injury.

Qualitative interview data strongly reinforced survey findings that care decisions after injury are considered a tradeoff between perceived severity and cost. Respondents established that (1) community members recognize the deleterious financial ramifications of using formal medical care; (2) perceptions of formal medical care and care choices are driven by prohibitive expense and cost structure; and (3) consequently, care decisions hinge on participant evaluations of severity. Although health care education was an important source of knowledge about injury, this finding is not generalizable to the larger study population; investigations to assess whether health education affects care-seeking behaviors in injured persons may be warranted.

A key finding of this study was the prohibitive cost of injury treatment and crippling financial losses incurred by Cameroonian families affected by injury. The total estimated expenditure for all injury treatment in the sample equates to US $5 626 000 per year in the Southwest Region alone. Notably, the mean cost of injury treatment was approximately half the cost of emergency treatment identified in a prior trauma registry–based study from Yaoundé, Cameroon.^4^ This difference likely reflects the community-based study design, which incorporates treatment costs from participants treated outside the formal care system. These data highlight the importance of considering disability as an independent metric when evaluating the cost of injury for policy makers.

Injury both impairs wage-earning capacity and often requires family members of the injured individuals to shift tasks to provide injury care. Projections from the 11 941 days of lost work in the study period equate to more than 2 million lost days of work per year in the region. Considerable rates of job loss, educational delay, and ongoing disability put affected individuals and households at high risk of long-term hardship and marginalization. Similar patterns identified in community-based data from Ghana suggest that catastrophic long-term disability is prevalent after injury throughout Sub-Saharan Africa.^20^

Use of formal medical care was associated with worsened economic outcomes. Although perhaps initially counterintuitive, this finding contextualizes the high rate of delayed or no formal medical care, even among communities where such care is readily available. Health financing schemes to decrease hardship and improve use of medical services after injury are urgently needed because previous work has suggested that cost is a considerable barrier to emergency care in LMICs.^21^

The financial burden of injury was also disproportionately great among participants from rural communities and among older participants. This disparity largely reflects the high costs of treatment among cohorts with lower baseline salaries, whereas financial losses owing to disability were similar between groups. These findings highlight that rural residents and older persons occupy particularly tenuous economic positions and are less able to compensate for the substantial financial effects of injury treatment costs and lost wages. Targeted efforts at secondary harm reduction, including public health education campaigns and government subsidization of care, should be deliberately inclusive of these communities.

Finally, our study highlights the critical disparity between the magnitude of injury as a public health concern and current prioritization of resources in LMICs. Historically, global health has incentivized treatment of infectious diseases but has largely ignored noncommunicable diseases, including injury. An increased global burden of noncommunicable diseases, however, has slowly shifted momentum toward developing capacity in these areas.^22,23^ Our data bolster growing evidence that investment of public health resources commensurate with the burden of injury are necessary to mitigate disability and spur economic development in Sub-Saharan Africa.

### Limitations

This study has several limitations. The community-based study design is inherently limited by participant memory and interpretation. Minor or intentional injuries are likely to be underreported, and estimates of disability duration and extent may not be precise.^11,24^ In addition, because some persons in resource-constrained settings may continue to work despite significant ongoing impairment, considering disability days as an isolated metric likely underestimates the morbidity associated with injury. The association between care seeking and disability may be confounded in the context of resource constraint, in which presentation to formal care does not always result in ideal medical management owing to cost, availability, or other barriers.

## Conclusions

This study suggests that injury presents a major public health problem to the Southwest Region of Cameroon and is associated with substantial postinjury disability and economic loss for affected families, communities, and the region. As in other reports from sub-Saharan Africa, we found that approximately one-third of injured persons failed to seek formal care after injury and that postinjury economic outcomes were substantially worse for the cohort who did seek formal medical care. The prohibitive cost of formal care in Cameroon may lead to care rationing, high poverty rates among affected families, and lost productivity for the region. To promote health and economic growth in the region, policy makers should focus attention on primary prevention of injury, including organizing public education campaigns and enforcing existing road safety policies, and consider the financial restructuring of emergency care using community-based health schemes and subsidization of care for vulnerable populations to increase access of trauma care to Cameroonians.

## Notes

Supplement. **eAppendix.** Community-Based Household Survey **eTable 1.** Household Demographic Comparison Between Community-Based Survey (2017) and Demographic and Health Survey (2011) Data for Southwest Region of Cameroon **eTable 2.** Factors Associated With Care-Seeking Decisions and Severe Financial Hardship After Injury **eTable 3.** Demographics, Injury Characteristics and Use of Care in Interviewed Households **eFigure.** Distribution of Disability Type and Severity After Injury in Southwest Region of Cameroon (n= 503)
