# Supplementary material for: Association of Health Care Use and Economic Outcomes After Injury in Cameroon
Source: JAMA Netw Open. 2020 May 19;3(5):e205171. doi: 10.1001/jamanetworkopen.2020.5171 (PMC7237963; doi:10.1001/jamanetworkopen.2020.5171)
Supplement: Supplement. — eAppendix. Community-Based Household Survey eTable 1. Household Demographic Comparison Between Community-Based Survey (2017) and Demographic and Health Survey (2011) Data for Southwest Region of Cameroon eTable 2. Factors Associated With Care-Seeking Decisions and Severe Financial Hardship After Injury eTable 3. Demographics, Injury Characteristics and Use of Care in Interviewed Households eFigure. Distribution of Disability Type and Severity After Injury in Southwest Region of Cameroon (n= 503) [file jamanetwopen-3-e205171-s001.pdf]

Supplementary Online Content

Christie SA, Dickson D, Mbeboh SN, et al. Association of health care use and economic outcomes after injury in Cameroon. *JAMA Netw Open*. 2020;3(5):e 205171. doi:10.1001/jamanetworkopen.2020.5171

eAppendix. Community-Based Household Survey

eTable 1. Household Demographic Comparison Between Community-Based Survey (2017) and Demographic and Health Survey (2011) Data for Southwest Region of Cameroon

eTable 2. Factors Associated With Care-Seeking Decisions and Severe Financial Hardship After Injury

eTable 3. Demographics, Injury Characteristics and Use of Care in Interviewed Households

eFigure. Distribution of Disability Type and Severity After Injury in Southwest Region of Cameroon (n= 503)

This supplementary material has been provided by the authors to give readers additional information about their work.

©2020 Christie SA et al. *JAMA Network Open*.

eAppendix. Community-Based Household Survey

**STUDY INFORMATION**	**Health District:**	**Health Area:**	**Interviewer Code:**	**Household Order:**	**Household #:**
**CONSENT STATUS**	☐ **Yes**<br>**Family Representative:** ___________________<br>**Family Name:**___________________________	☐ **No**<br>***If consent status switched during the survey was permission given to use previously collected data****?* *Yes / No*		☐ **Not Eligible**<br>*(Nobody >= age 18, not at home after multiple attempts, or cannot understand consent)*	

SECTION 1.1 Socioeconomics

1.1.1	**Is the household…**	☐ Rural/ More like a village? ☐ Urban/More like a city?
1.1.2	**Does any member of the household own a cellphone?**	☐ Yes ☐ No
1.1.3	**Does the household…**	☐ Own the home? ☐ Rent the home? ☐ Live for free?
1.1.4	**Does the household own agricultural land?**	☐ Yes ☐ No
1.1.5	**What types of cooking fuel are used in the household?** *(select all that apply)*	☐ Wood ☐ Charcoal ☐ Liquid Petroleum Gas (LPG) ☐ Kerosene ☐ Other
1.1.6a-b	a. **What is the highest education level achieved by any member of the household?**	☐ No formal education ☐ Primary School ☐ Secondary/High School ☐ Tertiary/College ☐ Unknown ☐ **Other**: **b.**_____________

SECTION 1.2 Household Members and Injury *(*Household= all persons who normally sleep under the same roof and share the same kitchen and including any such person who died in the last year)*

Starting with yourself, list the age and gender of each person living in your household* If anybody in the household died in the last year what was their age and gender?

Subject #	a.Age (years)	b. Sex (M/F)	c. If deceased, date of death? * (DD/MM/YYYY)	d. Were any of these people injured in the past year in a way that limited normal activities for at least one day or which needed treatment? **	e. On how many separate occasions was each person injured in the past year? (# separate occasions)	f. Does anybody in the household CURRENTLY have any of the following problems? *(write all applicable codes on the right for each person, IF THE* ***PROBLEM IS DUE TO INJUR****Y WITHIN 1 YEAR,* ***STAR****)*
1 (Respondent)						
2						
3						
4						
5						
6						
7						
8						
9						
10						
11						
12						
13						
14						
15						
16						
17						
18						

1.Breast changes like lumps, cancer, skin or nipple changes or discharge

2. Mass/swelling that changes size/sometimes disappears (HERNIA)

3. Other mass, lump or growth

4. Wound/Burn/scald

5. Deformity

6. Bleeding from rectum

7. New blindness or lost most of their vision

8. Abdominal distension OR severe/recurrent pain

19							9. Bleeding/sickness within 6 weeks of giving birth<br><br>10. Can't pass stool/urine or eat
20							
Study #s:	*If deceased, fill out Section 1.3, Cause of Death<br><br>**For each separate occasion for each injured person, fill out one Section 2 form. The TOTAL Occasions should equal the number of Section 2 forms needed				TOTAL ________________ Occasions **	TOTAL PROBLEMS __________ ***	***Fill out one Section 3 form for each separate problem- EXCLUDING those due to injury < 1 year ago (STARRED)

Section 1.3 CAUSE OF DEATH How many household members died in the past 1 year? _________

1.3.1a-d	*WRITE THE SUBJECT # OF EACH DECEASED FAMILY MEMBER IN THE GREY LEFT COLUMN AND FILL 3.2.1A-D*			
If death was due to an injury for which you will fill out a Section 2 select first option for 1.3.1a. You do not need to answer 3.2.1b-d for this person.				
**Write the Subject # of all household members who DIED in the past year**	**a. Did any of the following problems occur the WEEK before death or CAUSE the death?** *(select all that apply)*	**b. If any care was sought for this problem, where?** *(Circle all that apply)*	**c. Did they have an operation for the problem?**	**d. If not, what was the reason for NOT SEEING A DOCTOR or for NOT HAVING AN OPERATION?**
___________	☐ Wound/Injury in the past 1 year<br>☐ Wound (other)<br>☐ Mass, growth or swelling<br>☐ Deformity (they were born with)<br>☐ Deformity (they developed)<br>☐ Abdominal distention or pain<br>☐ Unable to pass stool/urine, or vomits all food<br>☐ Bleeding/sickness within 6 weeks of childbirth<br>☐ Abnormal appearing newborn<br>☐ None ☐ Unknown	***formal / traditional / home / other / none / NA***	***yes / no / unknown***	☐ No need<br>☐ No money<br>☐ No transportation/ Too far<br>☐ Facility, Personnel or Equipment not available<br>☐ No time<br>☐ Preference<br>☐ Unknown<br>☐ **Other:** _____________________
___________	☐ Wound/Injury in the past 1 year<br>☐ Wound (other)<br>☐ Mass, growth or swelling<br>☐ Deformity (they were born with)<br>☐ Deformity (they developed)<br>☐ Abdominal distention or pain<br>☐ Unable to pass stool/urine, or vomits all food<br>☐ Bleeding/sickness within 6 weeks of childbirth<br>☐ Abnormal appearing newborn<br>☐ None ☐ Unknown	***formal / traditional / home / other / none / NA***	***yes / no / unknown***	☐ No need<br>☐ No money<br>☐ No transportation/ Too far<br>☐ Facility, Personnel or Equipment not available<br>☐ No time<br>☐ Preference<br>☐ Unknown<br>☐ **Other:** _____________________
___________	☐ Wound/Injury in the past 1 year<br>☐ Wound (other)<br>☐ Mass, growth or swelling<br>☐ Deformity (they were born with)<br>☐ Deformity (they developed)<br>☐ Abdominal distention or pain<br>☐ Unable to pass stool/urine, or vomits all food<br>☐ Bleeding/sickness within 6 weeks of childbirth<br>☐ Abnormal appearing newborn<br>☐ None ☐ Unknown	***formal / traditional / home / other / none / NA***	***yes / no / unknown***	☐ No need<br>☐ No money<br>☐ No transportation/ Too far<br>☐ Facility, Personnel or Equipment not available<br>☐ No time<br>☐ Preference<br>☐ Unknown<br>☐ **Other:** _____________________
___________	☐ Wound/Injury in the past 1 year<br>☐ Wound (other)<br>☐ Mass, growth or swelling<br>☐ Deformity (they were born with)<br>☐ Deformity (they developed)<br>☐ Abdominal distention or pain<br>☐ Unable to pass stool/urine, or vomits all food<br>☐ Bleeding/sickness within 6 weeks of childbirth<br>☐ Abnormal appearing newborn	***formal / traditional / home / other / none / NA***	***yes / no / unknown***	☐ No need<br>☐ No money<br>☐ No transportation/ Too far<br>☐ Facility, Personnel or Equipment not available<br>☐ No time<br>☐ Preference<br>☐ Unknown

©2020 Christie SA et al. *JAMA Network Open*.

	☐ None ☐ Unknown			☐ **Other:** ______________________
__________	☐ Wound/Injury in the past 1 year<br>☐ Wound (other)<br>☐ Mass, growth or swelling<br>☐ Deformity (they were born with)<br>☐ Deformity (they developed)<br>☐ Abdominal distention or pain<br>☐ Unable to pass stool/urine, or vomits all food<br>☐ Bleeding/sickness within 6 weeks of childbirth<br>☐ Abnormal appearing newborn<br>☐ None ☐ Unknown	***formal / traditional / home / other / none / NA***	***yes / no / unknown***	☐ No need<br>☐ No money<br>☐ No transportation/ Too far<br>☐ Facility, Personnel or Equipment not available<br>☐ No time<br>☐ Preference<br>☐ Unknown<br>☐ **Other:** ______________________

©2020 Christie SA et al. *JAMA Network Open*.

SECTION 2: INJURY OCCASION

2.1 Subject #____________ Separate Occasion # _____*(1st occasion=1, 2nd occasion=2, etc.)*** **(NAME________________)**

**2.1.1**	**Date of Injury** *(approximate date if cannot remember)* ________________ DD/MM/YYYY

2.2 Pre-Injury Activities

**2.2.1**	**How many years of school has the injured person completed?** _____________years
**2.2.2a-c**	**a. In the 30 days prior to injury what job or activity did the injured person spend most of their time doing?** ____________________ ____________________ *When entering data, select occupation and industry based on this description and using the ECONOMIC CODE SHEET.* i. OCCCUPATION #______ ii.INDUSTRY #_____ Coder_______ **b. How many hours per week did they do this activity?** _________________**hours/week** **c.** About **how much did they earn doing this activity?** __________**CFA per** *(circle one)* **day / week / month / year**
**2.2.3**	**Did the person have another major job or activity?** □ Yes □ No □ Unknown

If "NO" or "UNKNOWN" skip to ***Section 2.3***

**2.2.4a-c**	**a. If yes, describe:** ____________________ ____________________ *When entering data, select occupation and industry based on this description and using the ECONOMIC CODE SHEET.* i. OCCCUPATION #______ ii.INDUSTRY #_____ Coder ______ **b. How many hours per week did they do this activity?** _________________**hours/week** **c. About how much did the person earn doing this activity?** __________**CFA per** *(circle one)* **day / week / month / year**

2.3 Injury Characteristics

**2.3.1a-b**	**a. What was the person doing when they became injured?** □ Work □ School □ Travel/Transit □ Sport □ Leisure/Play □ Unknown □ **Other:b.**_________
**2.3.2a-b**	**a. Where did the injury occur?** □ At home □ Someone else's home □ Street/highway □ Railway line □ Construction site □ Trade/Service area □ Farm □ Body of water □ Open land □ Sports area □ School □ Public area □ Unknown □ **Other:** **b.**______________
**2.3.3a-b**	**a. What caused the injury?** □ **Road Traffic Injury** □ Fall □ Blunt force (object/person) □ Blade/knife cut □ Firearm wound □ Other sharp injury □ Electrocution □ Burn from flame/heat □ Scald from hot liquid □ Smoke inhalation □ Explosive blast □ Poisoning □ Drowning □ Animal bite □ Unknown □ **Other:** **b.**______________

If "Road Traffic Injury" was NOT selected, skip to ***Question 2.3.5***

**2.3.4a-d**	**a. If Road Traffic Injury, what types(s) of transportation were involved?** *(select all that apply)* □ Pedestrian □ Private car □ Private motorcycle □ Taxi □ Mototaxi □ Minivan/minibus □ Truck/Lorrie □ Bus □ Train □ Bicycle □ Unknown □ **Other** **b.**__________ **c. If RTI, were seatbelts used?** □ Yes □ No □ NA **d. If RTI, were helmets worn?** □ Yes □ No □ NA
**2.3.5**	**Was the injury accidental or did somebody do this on purpose?** □ It was an accident (unintentional) □ Someone else did it to them on purpose □ They did it to themselves on purpose □ Unknown/Unsure
**2.3.6**	**Point to and tell me which part(s) of the body were injured…** *(select all that apply)* □ Head □ Face □ Neck □ Back at/above 12th rib □ Back below 12th rib □ Chest and torso □ Abdomen □ Pelvis □ Genitals □ Upper extremities □ Lower extremities □ Unknown
**2.3.7a-b**	**a. How was the person hurt?** (*Select all that apply)* □ Broken bone □ Sprain/strain □ Dislocation □ Cut/bite/wound □ Bruise or scrape □ Burn □ Poisoning □ Concussion/Brain injury □ Internal organ injury □ Pain (not otherwise specified) □ Unknown/unsure □ Other: **b.**______________________
**2.3.8**	**On the day of the injury, did the injured person?** *(Select all that apply)* □ Die □ Stop breathing □ Lose Consciousness □ Forget the injury □ Act confused □ None □ Unknown

©2020 Christie SA et al. *JAMA Network Open*.

2.4 Injury Treatment

2.4.1a-b

a. What type of person first provided help to the injured person?

- □ Family member
- □ Friend/ acquaintance
- □ Bystander/stranger
- □ Police officer
- □ Taxi/Mototaxi driver
- □ No help was provided
- □ Unknown
- □ **Other:** **b.**________________________________

2.4.2a-b

a. How was the injury treated? (*mark all that apply. Indicate the numeric order if known (1= first care type sought, 2= 2nd type sought…)*)

____No treatment sought ____Friend/acquaintance ____Traditional healer/ bonesetter ____ Unknown

____Family/ Home treatment ____Church ____Doctor/nurse/hospital/clinic ____ **Other b.**_____________

2.4.3

Approximately how much did all treatment cost (CFA)? _____________CFA

(Including outpatient costs, inpatient costs, laboratory fees, imaging fees, medicines, dressings, supplies, procedure fees, transport costs, and food costs above what the family would normally pay)

If HR gives itemized cost listing note below:

_____________CFA for

_____________CFA for

_____________CFA for

If formal care WAS sought FIRST skip to ***Question 2.4.5***

2.4.4a-b

a. If formal medical care (doctor /nurse…) was not sought FIRST which best describes why?

- □ Injury not serious
- □ Patient died before medical center was reached
- □ Too expensive
- □ No access to formal health services/ too far away
- □ Patient preference
- □ Unknown/Unsure
- □ **Other:b.**____

If formal care WAS NOT sought AT ALL skip to ***Section 2.5.***

When survey is completed, inform Qualitative RA household meets criteria for ***injured non-presenters****.*

2.4.5 **How many nights did the person stay in the hospital?** ____________________nights

2.4.6 **Did the patient receive an operation as part of their treatment?** □ Yes □ No □ Unknown

2.4.7a-b

a. Why did the injured person leave the hospital?

- □ They died
- □ They were sent home by staff (discharged)
- □ They went home against medical recommendation
- □ They were evaluated but never admitted
- □ Unknown/ Unsure
- □ **Other: b.** ____

2.4.8

Overall, how satisfied were you with the medical care your family member received?

- □ Satisfied
- □ Neutral
- □ Dissatisfied

2.4.9

Did your family experience any of the following while seeking formal care? *(select all that apply)*

- □ Long wait times
- □ Supplies or medicines unavailable
- □ Specialist unavailable
- □ Unable to pay for supplies, medicines, or care
- □ Rude or disrespectful treatment by staff
- □ Non- functioning equipment
- □ Poorly kept facilities

2.5 Mortality

If the injured person is STILL ALIVE (as indicated in Section 1), skip to ***Section 2.6***

2.5.1

How long after injury did the person die?

- □ Immediately
- □ <6 hours later
- □ 6- 24hours later
- □ >24 hours later
- □ Unknown

2.5.2

Would the person have died if they had not been injured?

□ Yes □ No □ Unknown/Unsure

2.6 Disability

2.6.1a-c

**a. Following injury did they have any NEW or WORSE difficulty with…** *(Select best option for each)*				**b. If yes, was the disability?** *(Circle)*	**c. Is this still a problem now?** *(Circle)*
**i. Speaking or communicating?**	___No	___Yes	___ Unknown	mild / moderate/ severe	yes / no / unknown
**ii. Dressing, eating, going to the bathroom?**	___No	___Yes	___ Unknown	mild / moderate/ severe	yes / no / unknown
**iii. Leaving the home, shopping, traveling?**	___No	___Yes	___ Unknown	mild / moderate/ severe	yes / no / unknown
**iv. Engaging with friends/ family?**	___No	___Yes	___ Unknown	mild / moderate/ severe	yes / no / unknown
**v. Going to school?**	___No	___Yes	___ Unknown	mild / moderate/ severe	yes / no / unknown
**vi. Seeing or hearing?**	___No	___Yes	___ Unknown	mild / moderate/ severe	yes / no / unknown
**vii. Standing or walking?**	___No	___Yes	___ Unknown	mild / moderate/ severe	yes / no / unknown
**viii. Picking things up or using their hands?**	___No	___Yes	___ Unknown	mild / moderate/ severe	yes / no / unknown
**ix. Weakness, shortness of breath, fatigue?**	___No	___Yes	___ Unknown	mild / moderate/ severe	yes / no / unknown
**x. Understanding or remembering things?**	___No	___Yes	___ Unknown	mild / moderate/ severe	yes / no / unknown
**xi. Depression or shame?**	___No	___Yes	___ Unknown	mild / moderate/ severe	yes / no / unknown

©2020 Christie SA et al. *JAMA Network Open*.

2.6.2	**Did the injured person lose their job or completely stop going to school because they had been injured?**<br>□ Yes □ No □ Unknown □ NA
2.6.3a-d	**a. Has the patient been readmitted to the hospital or needed additional treatment for problems related to their injury?**<br>*(NOT including scheduled follow-up visits)*<br>□ **Yes** □ No □ Unknown<br>**es, how many..**<br>_______ **re-admissions?**<br>_______ **consultations/ visits?**<br>_______ **operations?**
2.6.4	**Will the injured person likely need further medical care in the future for problems related to their injury?**<br>□ Yes □ No □ Unknown

2.7 Economic Impact

2.7. 1	**After being hurt, was there at least one day the injured person was not able to perform their main job/ activity by themselves?**<br>□ Yes □ No □ Unknown
	If "NO" or "UNKNOWN" skip to ***Question 2.7.3***
2.7.2 a-c	**a. If yes, for how many days?** __________days<br>**b. How much of the job/activity was completed by others?** □ All of it □ Most of it □ Some of it □ Unknown/NA<br>**c. Who performed or helped them perform the job/activity?** □ Family □ Non-family □ Nobody □ Unknown/NA
	If the injured person DID NOT have a second major job or activity (Question 2.2.3), skip to ***Question 2.7.5***
2.7.3	**After being hurt, was there at least one day the injured person could not perform their secondary job/activity by themselves?**<br>□ Yes □ No □ Unknown
	If "NO" or "UNKNOWN" skip to ***Question 2.7.5***
2.7.4a-c	**a. If yes, for how many days?** ___________days<br>**b. How much of the job/activity was completed by others?** □ All of it □ Most of it □ Some of it □ Unknown/NA<br>**c. Who performed or helped them perform the job/activity?** □ Family □ Non-family □ Nobody □ Unknown/NA
2.7.5a-b	**a. After being hurt, was there even one day another family member had to shift their usual activities to care for the injured person (CARETAKER)?**<br>□ **Yes** □ No □ Unknown<br>**b. If yes, for how many days?** ______days
	If "NO" or "UNKNOWN" skip to ***Question 2.7.9***
2.7.6a-c	**a. In the 30 days prior to the person's injury what job/activity did the CARETAKERS spend most of their time doing**_________________________________**?**<br>*When entering data, select occupation and industry based on this description and using the ECONOMIC CODE SHEET.*<br>i. OCCCUPATION #______ ii. INDUSTRY #_____ Coder_______<br>**b. How many hours per week did they do this activity?** _________________**hours/week**<br>**c. About how much did the person earn doing this activity?** ___________**CFA per** *(circle one)* **day / week / month / year**
2.7.7	**In the 30 days prior to the injury, did the CARETAKER have another major job/activity?**<br>□ Yes □ No □ Unknown
	If "NO" or "UNKNOWN" skip to ***Question 2.7.9***
2.7.8a-c	**a. If yes, describe:**________________________________<br>____<br>*When entering data, select occupation and industry based on this description and using the ECONOMIC CODE SHEET.*<br>i. OCCCUPATION #______ ii. INDUSTRY #_____ Coder_______<br>**b. How many hours per week did they do this activity?** _________________**hours/week**<br>**c. About how much did the person earn doing this activity?** ___________**CFA per** *(circle one)* **day / week / month / year**
2.7.9	**Following the injury, did it become more difficult for the family to afford expenses such as food and rent?**<br>□ Yes □ No □ Unknown
2.7.10a-d	**a. Following the injury did the family do any of the following**? *(select all which apply)*<br>□ Sell assets (livestock, vehicles, property etc.) — b. **If so, how much was made from the sale?** _____________CFA<br>□ Spend saved money — c. **If so, how much was spent?** ____________CFA<br>□ Borrow money — d. **If so, how much was borrowed?** _____________CFA<br>□ None<br>□ Unknown
2.7.11	**How long do you expect the injured person's new limitations to last?**<br>□ They recovered □ ________ *(circle one)* **day / week / month / year** □ Forever □ Unknown

©2020 Christie SA et al. *JAMA Network Open*.

Section 4: Opinions of Formal Care and Health Knowledge Assessment

**4.1**	**Where would your family most likely go for medical care after injury?** ___________________________
**4.2**	**In your opinion, what is the biggest problem with formal medical care services in Southwest Cameroon?**<br>□ Too expensive<br>□ Too far away/difficult to reach<br>☐ The treatments don't work<br>□ Rude behavior by staff<br>□ Traditional medicine is preferred<br>☐ Faith treatment is preferred<br>☐ None<br>□ **Other: ii.**____
**4.3 a-b**	**a. Do you know how to get to the nearest health facility?** □ Yes □ No<br>**b. If "Yes" what type of transportation would you most likely use to travel there?** __________________________<br>**c. Using this method, approximately how long do you think it would it take to travel there?** _______hours
**4.4**	**Which of the following conditions do you believe can be treated or reversed with surgery?** □ None<br>*(select all that apply)*<br>□ Some types of blindness □ Some types of cancer □ Injury □ Burns □ Deformities
*If the respondent is **MALE** please skip to **Section 5***	
**4.5 a-c**	**Do you know how to perform breast self-exam?**<br>□ No, I've never heard of it □ No, I have heard of it but don't know how to do it □ Yes, I know how to do it
*If "No…" was selected, skip to **Section 5***	
	b. **How often have you done breast self-exam in the past year?**<br>□ Monthly<br>□ Several times<br>□ Once<br>□ None<br>□ I have NEVER done it
	c. **What time of the month do you perform self-breast exam?**<br>□ At any time<br>□ First week after menses<br>□ Second week after menses<br>□ Menses

Section 5: LANGUAGE BARRIERS IN FORMAL HEALTHCARE

**5.1a-c**	**a. What is your region of origin?** □ Southwest, Cameroon<br>□ **Other region in Cameroon** b. **Specify:**__________________________<br>□ **Other country** c. **Specify country:**______________
**5.2a-b**	**a. Which language is spoken by the household most often at home?**<br>□ Pidgin English □ English □ French □ **Local language or other: b.**_______
**5.3a-b**	**a. Which other languages are spoken by household members ?** *(select all that apply)*<br>□ Pidgin English □ English □ French □ **Local language or other: b.**_______
**5.4**	**If you felt a doctor did not understand you, what would you most likely do?**<br>□ Find another patient or family member to help translate<br>□ Follow instructions to my best ability using what I do understand<br>□ Find a different doctor<br>□ Leave and go to a traditional healer<br>□ Leave and treat the problem at home<br>□ **Other: b.**______________________________
**5.5**	**Has anyone in the household ever gone to a hospital/medical center for any reason?**<br>□ Yes □ No □ Unknown
If "Yes" ask questions 5.5 – 5.9. If "No" or "Unknown" end survey	
**5.6a-b**	**a. If yes, what languages were spoken with staff?** *(select all that apply)*<br>□ Pidgin English □ English □ French □ **Local language or other: b.**_____________
**5.7a-c**	**How much did the household member(s) understand:** *(For each letter circle best answer)*<br>**a. what the doctor told them about the problem?** All of it / Most of it / Some of it / Very little / None of it / Unknown / NA<br>**b. instructions for how to take medicines correctly?** All of it / Most of it / Some of it / Very little / None of it / Unknown / NA<br>**c. instructions for caring for the problem after returning home?** All of it / Most of it / Some of it / Very little / None of it / Unknown / NA
**5.8**	**Did the hospital staff understand what the household member(s) said to them?** *(circle best answer)* All of it / Most of it / Some of it / Very little / None of it / Unknown
**5.9**	**Has anyone in the household brought another person to the hospital/medical center to help them translate?**<br>□ Yes □ No □ Unknown

©2020 Christie SA et al. *JAMA Network Open*.

eTable 1. Household Demographic Comparison Between Community-Based Survey (2017) and Demographic and Health Survey (2011) Data for Southwest Region of Cameroon

		Community-Based Survey (2017) *n= 8065*	Demographic and Health Survey (2011)[a]
		Mean ± Std. Err. or Percentage	Mean ± Std. Err. or Percentage
**Age**		23.9 ± 0.2	22.2 ± 0.3
**Gender**			
	Male	48%	48%
	Female	52%	52%
**Household owns cellphone**		95%	77%
**Household members**		6.3 ± 0.2	6.0 ± 0.1
**Household owns agricultural land**		65%	66%
**Household home ownership**			
	Owns the home	63%	57%
	Rents the home	25%	32%
	Lives for free	11%	11%
**Urban**[b]		*58%*	*44%*
**Petroleum gas fuel used**[c]		*43%*	*18%*
**Wood fuel used**[c]		*92%*	*81%*

[a] *Data from 2011 Cameroon Demographic and Health Survey (DHS)(11)*
[b] *Cameroonian Community Based Survey defined urban as multiple tarred road access whereas DHS data reports population size over 10,000.*
[c] *Cameroonian Community Based Survey asked about all cooking types used in the home whereas DHS data reports the most prevalent cooking type used by the household.*

©2020 Christie SA et al. *JAMA Network Open*.

eTable 2. Factors Associated With Care-Seeking Decisions and Severe Financial Hardship After Injury

	**Adjusted Odds Ratio**	**95% CI**
**Delayed or Non-Presentation**		
Age	0.99	0.98 -1.00
Injury severity	0.37	0.21 -0.67
Blunt trauma mechanism	2.42	1.33 - 4.41
Fall	2.42	1.52 -3.84
Absence of first responder	1.92	1.18 - 3.13
**Formal Care Use**		
Age		
Injury severity at scene		
Road traffic injury	2.17	1.24 - 3.78
Bystander first responder	2.65	1.25 - 5.60
Injury sustained to torso	4.61	1.53 - 13.95
Injury sustained to head & neck	2.41	1.33 - 4.39
**Severe Financial Hardship**		
Road traffic injury	2.13	1.29 -3.53
Formal care use	1.67	1.05 -2.65
Disability days	1.02	1.01 -1.02
Age, per 20 years	1.35	1.04 – 1.76
Caretaker needed	1.01	0.99 – 1.03
Urban household	0.47	0.29 -0.74
Treatment cost, per US$20	1.13	1.05 -1.21

Legend: Multivariate logistic regression models were built using a stepwise regression analysis process.

©2020 Christie SA et al. *JAMA Network Open*.

eTable 3. Demographics, Injury Characteristics and Use of Care in Interviewed Households

Interview	Age	Gender	Region	Mechanism	Severity	Care Type(s)
**1**	Adult	F	Urban	RTI	Moderate	Formal Care
**2**	Adult	F	Urban	RTI	Severe	Formal Care
**3**	Adult	M	Semi-Rural	Burn	Minor	Informal Care
**4**	Child	M	Urban	RTI	Moderate	Formal Care
**5**	Child	M	Urban	RTI	Moderate	Multiple Care
**6**	Child	F	Semi-Rural	RTI	Moderate	Multiple Care
**7**	Adult	M	Rural	RTI	Minor	Multiple Care
**8**	Adult	F	Rural	Fall	Severe	Multiple Care
**9**	Adult	M	Rural	Blade/Knife	Moderate	Multiple Care
**10**	Adult	M	Urban	Blunt (other)	Severe	Multiple Care
**11**	Child	M	Semi-Rural	Fall	Severe	Informal Care
**12**	Adult	F	Urban	Burn	Moderate	Informal Care
**13**	Adult	F	Urban	Fall	Severe	Informal Care
**14**	Child	F	Urban	RTI	Severe	Multiple Care
**15**	Adult	F	Urban	RTI	Severe	Multiple Care
**16**	Child	F	Semi-Rural	RTI	Severe	Death Prior to Care
**17**	Adult	F	Rural	RTI	Severe	Informal Care
**18**	Adult	F	Rural	Burn	Severe	Formal Care
**19**	Adult	M	Urban	RTI	Severe	Multiple Care
**20**	Adult	M	Urban	RTI	Moderate	Informal Care
**21**	Adult	F	Urban	RTI/Fall	Moderate	Multiple Care
**22**	Child	M	Rural	Burn	Moderate	Multiple Care
**23**	Child	M	Rural	Sharp	Moderate	Formal Care
**24**	Adult	F	Rural	Fall	Severe	Informal Care
**25**	Child	F	Rural	Fall	Moderate	Informal Care
**26**	Adult	F	Rural	Fall	Severe	Death Prior to Care

©2020 Christie SA et al. *JAMA Network Open*.

**27**	Adult	M	Semi-Rural	Blunt	Severe	Multiple Care
**28**	Adult	M	Rural	Fall	Moderate	Formal Care
**29**	Adult	M	Rural	Assault	Severe	Multiple Care
**30**	Adult	M	Rural	RTI	Severe	Formal Care
**31**	Adult	M	Semi-Rural	RTI	Severe	Formal Care
**32**	Child	M	Urban	Blade/Knife	Moderate	Informal Care
**33**	Adult	M	Urban	Assault	Severe	Formal Care
**34**	Adult	F	Urban	RTI	Moderate	Formal Care

Legend: Adult, >18 years old; Child, <18 years old; F, Female; M, Male; RTI, Road Traffic Injury; Minor, injury likely to lead to minimal to no disability regardless of care type sought; Moderate, untreated injury likely to lead to significant permanent disability or complications; Severe, life or limb threatening injury. Age and gender are reported for injured subject; respondent may be of a different age/gender. Severity designations assigned by authors on the basis of injury descriptions provided in interviews.

©2020 Christie SA et al. *JAMA Network Open*.

eFigure. Distribution of Disability Type and Severity After Injury in Southwest Region of Cameroon (n= 503)

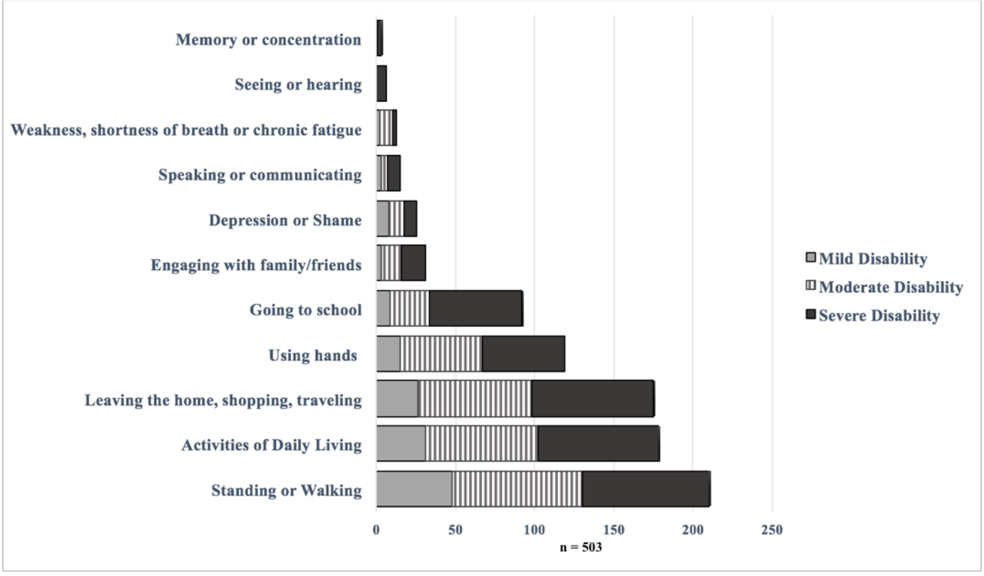

©2020 Christie SA et al. *JAMA Network Open*.
